# Management of Polymicrobial Cierny-Mader Grade 3 and 4 Chronic Osteomyelitis of the Femur

**DOI:** 10.7759/cureus.12818

**Published:** 2021-01-20

**Authors:** Zaki Arshad, Aiman Aslam, Edward Lau, Azeem Thahir, Matija Krkovic

**Affiliations:** 1 Trauma and Orthopaedic Surgery, University of Cambridge School of Clinical Medicine, Cambridge, GBR; 2 Trauma and Orthopaedic Surgery, Addenbrooke's Hospital, Cambridge University Hospitals NHS Foundation Trust, Cambridge, GBR

**Keywords:** bone infection, femur, health economics

## Abstract

Introduction

Osteomyelitis refers to an inflammatory process affecting the bone and bone marrow. Chronic disease occurs following the formation of a necrotic, devascularised sequestrum. Regardless of the approach, treatment is complex and resource-intensive, often requiring multiple surgical interventions and extended antibiotic therapy. This study aims to review the treatment of chronic osteomyelitis of the femur by a single surgeon over seven years (January 2013 to January 2020).

Materials and methods

We retrospectively reviewed a consecutive series of 14 patients. Data collected includes age, sex, medical history, treatment, pathogen, C-reactive protein levels, outcome, and follow-up period. The EuroQOL five-dimensional questionnaire (EQ-5D-5L) and Visual Analogue Scale (EQ-VAS) were used to assess health outcomes. Data concerning total treatment costs and reimbursement received was also collected.

Results

Although 10/14 (71.4%) patients were considered in remission at final follow-up, only six (42.9%) achieved remission and showed no recurrence after initial treatment. The mean total treatment cost was £39,249.50, with a mean deficit of - £19,080.10 when considering reimbursement. Patients showed a significantly lower mean EQ-5D score (0.360) compared to the national population mean of 0.856 (P = .0018) as well as a lower mean EQ-VAS (61.7) compared to the population norm of 82.8 (P = .013).

Conclusion

The extensive nature of the infection, high rates of co-morbidity, and the growth of more than a single pathogen may explain the lower success rate observed. In these patients, recurrence may be highly likely and thus regular follow-up is vital in order to ensure effective management.

## Introduction

Osteomyelitis refers to inflammation of the bone and bone marrow due to infective causes, resulting in progressive inflammatory destruction of local bone architecture and subsequent necrosis and apposition of new bone [1]. The pathophysiology of the condition can be broadly split into three general categories: haematogenous spread, contiguous contamination, and infection due to neurovascular insufficiency, as in diabetes mellitus [1]. In the case of chronic infection, inflammatory exudate deposition leads to an increase in intramedullary pressure, periosteal stripping, and vascular thrombosis. The subsequent lack of vascular supply results in the formation of a necrotic area of bone, known as a sequestrum. The de-vascularised nature of this area prevents the clearance of pathogens and antibiotic penetration, leading to chronic osteomyelitis [2].

Managing chronic osteomyelitis remains a complex process, often requiring a multi-stage intervention including debridement, accurate pathogen identification, osseous fixation, management of dead-space, and a combination of appropriate local and systemic antibiotic therapy [3]. Whilst studies have shown favourable results with the use of a radical debridement strategy, the extent of bony resection must be carefully balanced with the need for subsequent defect management and ensuring structural stability. Aside from procedure complexity, management is resource and service-intensive due to the high rates of complication and involvement of senior colleagues from various disciplines, such as plastic surgery and infectious diseases.

Together, these factors contribute to the significant economic burden associated with treating osteomyelitis, regardless of whether treatment is in a specialist centre or elsewhere [4]. The disparity between the cost of chronic osteomyelitis management and remuneration, according to the Healthcare Resource Group (HRG) code, has been reported at an Oxford-based centre, where a mean deficit of £10,437.6 across 60 cases was described [5]. However, generally, there is a paucity of similar information from individual centres across the country.

Herein, we present a single-surgeon case series of management of chronic osteomyelitis of the femur at our tertiary centre based in the United Kingdom (UK) over the seven-year period between January 2013 and January 2020. We aim to assess treatment outcomes in terms of infection eradication and complications such as infection recurrence or requirement for further treatment. We also aim to understand the economic implications of treatment, considering management costs against funding received from the local clinical commissioning group (CCG).

## Materials and methods

This study was conducted as part of a service evaluation, approved by the hospital. Using the hospital electronic patient record system, we retrospectively reviewed the personal logbook of the senior author (MK) for patients treated for chronic osteomyelitis of the femur between January 2013 and January 2020. Patients were classified according to the Cierny-Mader (CM) and bone involvement, antimicrobial options, soft tissue coverage and host status (BACH) classification systems. The Cierny-Mader classification scheme includes an assessment of both the extent of bony involvement and host condition, whilst the BACH scheme also takes into account patient antibiotic sensitivity and wound closure difficulty [6,7].

All patients received a combination of various surgical treatments including sequestrectomy, reamer-irrigation-aspiration (RIA), debridement, vacuum-assisted closure (VAC), washout, and bone grafting. Calcium sulphate beads loaded with vancomycin and gentamycin were implanted in all cases. At least five intra-operative samples were taken per patient, and microbiology results were recorded. Identification of the specific pathogen was used to guide the administration of targeted intravenous antibiotic therapy for a minimum duration of six weeks following initial surgery. All operations were performed by the same surgeon (MK), who also provided post-operative outpatient follow-up and clinical examination, along with a member of the centre’s infectious diseases team. Serial biochemical blood tests including C-reactive protein (CRP) and erythrocyte sedimentation rate (ESR) were performed in all cases. Remission was defined as the absence of clinical signs of osteomyelitis such as sinus discharge and CRP <4 mg/L, which was the upper limit of our lab’s reference range. 

Using the patient-level information and costing system (PLICS), data regarding the costs associated with treating patients’ osteomyelitis including operation, medication, ward stay, staff, and follow-up costs were collected. Data on the amount of money our centre received from the local CCG to treat individual patients were also collected, using which we were able to calculate a net profit/loss associated with treatment.

After the latest clinic follow-up appointment, attempts were made to contact all patients via telephone in order to assess functional outcomes using the EuroQOL five-dimensional interviewer administration questionnaire (EQ-5D-5L™) and the EuroQOL visual analogue scale (EQ-VAS™). Patients who were unreachable after four calls on four different days, at different times of the day, were excluded from this part of the study. Informed consent was obtained from all included patients. The current National Institute for Health and Care Excellence (NICE) position statement recommends that the EQ-5D-5L value set for England outline by Devlin et al. should not be used to derive index values due to methodological concerns [8]. Therefore, we derived index values by mapping EQ-5D-5L data onto the EuroQOL three-dimensional questionnaire (EQ-5D-3L) value set using the EuroQOL crosswalk index value calculator, based on the function developed by Van Hout et al. [9].

IBM Statistical Package For the Social Sciences (SPSS) Statistics 25 (IBM Corp., Armonk, NY) was used to compare questionnaire results to established population norms. Welch’s t-test was chosen due to the large difference in sample size between our study and that used to derive population norms. Here, a value of P< .05 was considered to be significant.

## Results

A total of 14 consecutive patients were identified and included on the basis of clinical and radiographic signs of chronic osteomyelitis, such as the presence of a discharging sinus and/or a radiographically visible sequestrum or periosteal reaction. The mean age at initial presentation was 47.9 years (range: 30-61), with 11 male and three female patients included. All except one of the patients displayed one or more osteomyelitis risk factors, such as a history of smoking, intravenous drug use, type two diabetes, or immune-compromised. A history of femoral fracture after a road traffic accident or mechanical fall was common, with 11 patients having previously received internal fixation for such an injury. History of cellulitis and thigh abscess was reported in one patient, whilst another had previously received extensive surgical treatment for necrotizing fasciitis and a further patient had previously undergone knee fusion due to synovial osteochondromatosis. All patients were classified as suffering from either grade 3 or 4 chronic osteomyelitis according to the Cierny-Mader classification system (Table 1). 

**Table 1:** The cases of chronic osteomyelitis treated at our centre in the described time period. CM = Cierny-Mader, LOS = length of hospital stay, M = male, F = female, RIA = reamer-irrigator-aspirator, IVDU = intravenous drug user, VAC = vacuum-assisted closure, CRP = C-reactive protein, RTA = road traffic accident, T2DM = type two diabetes mellitus

Number	Age (Gender)	History	Co-morbidities	BACH/CM	Microbiology	Surgical Treatment	Outcome	Complications (Follow-up period in months)	LOS (Days)
1	54 (M)	RTA: femoral fracture with nailing. Osteomyelitis previously treated with RIA and debridement.	Smoker	B2A2C1H2/4B	Paenibacillus macerans, Enterobacter cloacae	RIA + debridement with multiple washouts and application of cement (gentamycin + vancomycin). Cement removed and bone graft applied.	Remission	None (26)	55
2	61 (M)	Femoral shaft fracture with nailing. Healed 4cm short, lengthening with external fixation.	45 pack-year smoker, gout	B1A1C1H1/3B	Staphylococcus aureus	Removal of metalwork + RIA	Remission	None (24)	10
3	43 (F)	Cellulitis + abscess. Debridement with multiple washouts.	IVDU, hepatitis C	B2A1C1H2/4B	Staphylococcus aureus, Candida tropicalis, Candida glabrata	Multiple debridements and washouts + VAC	Lowest CRP 20mg/L	Readmitted with fracture after mechanical fall and recurrence of infection. External fixation applied with further antibiotic therapy. Remission achieved. (27)	111
4	45 (M)	RTA: femoral fracture with nailing. Nail removed due to infection.	IVDU, smoker (nicotine + cannabis), controlled hepatitis C	B1A2C1H1/3B	Citrobacter koseri, Enterobacter cloacae	Debridement + multiple washouts + VAC	Lowest CRP 41 mg/L but no clinical signs of infection	None (7)	83
5	43 (M)	RTA: distal femur fracture with nailing.	Smoker, gout, sarcoid	B2A1C1H1/4B	Staphylococcus epidermis, Staphylococcus aureus	Sequestrectomy + nail removal + bone transport over a long intramedullary nail	Remission	Recurrence of infection. Further resection of femur and bone transport over IM nail and antibiotics. Remission achieved. (29)	40
6	54 (M)	Synovial osteochondromatosis with knee fusion and synovectomy. Debridement and curettage of tibial sinus.	None	B2A1C2H1/4A	Escherichia coli, Proteus mirabilis	RIA + multiple debridements + sequestrectomy + VAC + bone transport with Taylor Spatial Frame.	CRP constantly elevated (lowest 18 mg/L).	Consistent signs of infection at subsequent follow-up. Given above-knee amputation after which remission achieved. (49)	95
7	44 (M)	RTA: femoral fracture with nailing. Nail removed.	20 pack-year smoker, T2DM	B1A1C1H1/3B	Staphylococcus aureus	RIA + debridement	Remission	Recurrence - thigh incision and drainage with VAC and targeted antibiotics. Remission achieved. (27)	23
8	43 (F)	Femoral fracture and nailing (Non-union). Drainage of abscess and application of external fixator.	IVDU, 25 pack-year smokers.	B2A2C1H1/4B	Proteus mirabilis, Corynebacterium tuberculostearicum, Candida sp., Stenotrophomonas maltophilia, vancomycin-resistant Enterococcus	RIA + multiple debridements and VAC + change of external fixator	Lowest CRP 44 mg/L	Readmitted with pin site infection, given targeted oral antibiotics, CRP reduced to 21 mg/L (19)	109
9	52 (M)	Open femoral shaft fracture with nailing and stage one Masquelet procedure.	Daily smoker	B2A2C1H1/4B	Staphylococcus epidermis, Staphylococcus hominins	Washout + debridement + VAC + application of external fixation	Lowest CRP 9 mg/L	Given revision nailing and further debridement and VAC. CRP reduced to 14mg/L with no clinical signs of infection (36)	74
10	59 (M)	RTA: femoral fracture with nailing. Multiple previous osteomyelitis flare-ups treated with antibiotic therapy and washout.	T2DM	B2A1C1H2/4B	Staphylococcus schleiferi, Paenibacillus macerans	RIA + debridement + sequestrectomy	Remission	None (14)	7
11	49 (M)	Compound femur fracture with external fixation and nailing. Multiple debridements and washouts.	20 pack year smoker, gout	B1A1C1H2/3B	Corynebacterium sp., Actinobaculum	RIA + sequestrectomy	Remission	None (9)	4
12	39 (M)	Necrotising fasciitis with split skin graft and debridement.	IVDU, 20 pack year smoker	B1AXC1H1/3B	No growth	RIA + debridement + sequestrectomy	Remission	None (17)	26
13	55 (F)	Femoral shaft fracture and nailing.	Smoker	B2A2C1H1/4B	Prevotella loescheii, Eikenella corrodens	Multiple debridements + VAC + insertion of intramedullary nail	Lowest CRP of 18, no clinical signs of infection	None (7)	31
14	30 (M)	RTA: femoral shaft fracture and nailing.	Splenectomy	B2A1C1H1/4B	Staphylococcus epidermis, Pseudomonas aeruginosa, Paenibacillus sp.	RIA + sequestrectomy + external fixation	Remission	None (8)	15

A wide range of pathogens was identified (Table 1), with the most common pathogens being *Staphylococcus aureus* (three patients), *Staphylococcus epidermidis* (three patients), and Paenibacillus sp. (three patients). Most patients (11/14, 78.6%) demonstrated polymicrobial infection, whilst no bacterial growth was identified in only one patient. This patient did receive antibiotic therapy prior to culture testing, which may explain the negative result. The mean cumulative length of inpatient hospital stay was 48.9 days (range: 4-111).

Clinical remission was achieved after initial treatment in eight patients (57.14%), with six of these presenting no signs of recurrence during the follow-up period. The remaining two patients required further surgical and targeted antibiotic therapy in order to achieve remission after a period of recurrence. In six patients, CRP levels did not reduce sufficiently (<4 mg/L) after initial treatment to consider them to be in complete clinical remission at that point. Out of these six, four patients received further surgical/antibiotic therapy, after which remission was achieved in one patient with a second patient achieving remission only after having a subsequent above-knee amputation, and two further patients achieved a substantial reduction in CRP. The remaining two of these six patients failed to show complete biochemical remission after initial treatment and received no further treatment during the follow-up period due to a lack of concerning clinical symptoms. The mean follow-up period was 21.4 months (range: 7-49).

A total of nine patients were contactable and were able to complete the EQ-5D-5L questionnaire (Table 2). The mean derived EQ-5D-3L index value score was 0.360 (standard deviation = 0.324), which is significantly lower than the UK population norm of 0.856 reported by Szende et al. (t(8)= 4.59, P = .0018) [10]. Similarly, a significant difference was also found between our EQ-VAS mean of 61.6 and the reported population norm of 82.8 (t(8)=3.18, P = .0130).

**Table 2:** Results of the EQ-5D-5L questionnaire

	Mobility, n	Self-care, n	Usual Activities, n	Pain/Discomfort, n	Anxiety/Depression, n
Level 1 (no problems)	2	2	1	2	3
Level 2 (slight problems)	0	3	1	0	4
Level 3 (moderate problems)	4	3	2	3	1
Level 4 (severe problems)	1	1	3	4	1
Level 5 (extreme problems/unable to do)	2	0	2	0	0

Patient-level costing information was available for four members of the reviewed cohort. The mean cost per patient was £39,249.50 (range: £18,317.80-60,534.10). Upon closer inspection, ward costs were the biggest cost in all of these cases, accounting for 39.7% of the cumulative treatment costs across the four patients. Other major contributors were theatre costs, theatre staffing, and devices and prostheses, accounting for 15.7%, 15.2%, and 10.0%, respectively. When considering income received from the local CCG for these patients, a negative margin was seen in all cases, with a mean loss of £19,080.10 (range: £5,504.20-28,030.80).

We were also able to retrieve patient-level costing information for a separate cohort of 20 patients treated at our centre, for chronic osteomyelitis, between 2018 and 2019. This cohort demonstrated a similar financial burden to our primary study cohort, with a mean cost per patient of £35,475 and a mean negative margin of £21,414.60.

## Discussion

Chronic osteomyelitis is difficult to manage due to the presence of extensive devascularised bone and the formation of a bacterial biofilm during chronic inflammation [11,12]. This biofilm consists of a sessile community of bacteria attached to either an interface or each other and embedded in the bone matrix. This protective structure may confer resistance to both antibiotic therapy and host immune clearance, through reduced levels of bacterial division and the formation of a diffusion barrier [12,13]. Furthermore, biofilms may facilitate dispersion of bacteria following surgical debridement, leading to recurrence after initial treatment [12].

Although the specific treatment regime used in our series varied between individual patients, the same four principles were used to guide treatment in all cases: (1) debridement/sequestrectomy/reaming of infected areas, (2) local antibiotic delivery via calcium sulphate beads, (3) identification of a causative pathogen, and (4) targeted systemic antibiotic therapy where possible. Various slightly different techniques based around the same four principles are reported in the existing literature. For example, Kanakaris et al. describe the use of an RIA system, along with the insertion of antibiotic-loaded cement rods, reporting that 95.8% of patients achieved remission and showed no further signs of recurrence during the follow-up period [14]. A similarly high success rate of 96.7% is reported by Gokalp et al. with the use of a technique involving the creation of a bone “gutter” through the removal of infected tissue and muscle flap transposition [15]. The use of bioactive glass is also reported, with Drago et al. showing a success rate of 88.9% through debridement and insertion of bioactive glass granules [16]. Both Egol et al. and Huang et al. describe a treatment strategy involving debridement, sequestrectomy, and targeted antibiotic therapy, with the latter also using the placement of antibiotic-impregnated polymethylmethacrylate (PMMA) bone cement [17,18]. Despite the similarity in treatment strategy between these two studies, a large difference in success rate is evident, with Egol et al. reporting that 84.4 % of patients achieved remission with no further recurrence during follow-up, whilst this figure lies only at 50% in the series of Huang et al. 

The differences in success rate reported above may be down to factors such as differences in follow-up time, type and number of causative pathogens, and length of antibiotic therapy. Additionally, the success rate may be influenced by the severity of disease, however, the lack of use of Cierny-Mader or BACH classification systems amongst many studies makes it difficult to compare the severity of diseases across studies. Furthermore, there is no universal criteria used to define remission or recurrence in previous studies. For example, Huang et al. use a strict CRP cut-off value of 5mg/L as well as clinical symptoms and radiographic results to define remission [18]. Meanwhile, Egol et al. use a CRP cut-off value of 10mg/L together with the lack of a draining sinus, and both Kanakaris et al. and Drago et al. use a combination of unspecified CRP values, clinical signs, and radiography [14,16,17]. Additionally, previous studies include treatment of chronic osteomyelitis in a range of long bones including the tibia, femur, humerus, radius, and others, often failing to provide a breakdown of results according to the bone affected. It is possible that differences in the exact bones affected in included studies could also contribute to varying treatment success.

Although 71.4 % of our patients were considered in remission at final follow-up, only 42.9% achieved remission after initial treatment and did not show signs of recurrence during follow-up; this value is lower than in similar studies. However, given that all included patients showed signs of more severe (Cierny-Mader grade 3 or 4) disease and all except one showed risk factors such as a history of smoking and diabetes, this is perhaps expected. For instance, Egol et al. report a higher initial success rate however over half of the cases (56%) included were graded 2B or under according to the Cierny-Mader classification, with only 32% of cases being of grade 3 or 4 [17]. Another possible factor is the number of causative pathogens, with 11/14 patients in our series showing the growth of more than one pathogen. Almost half (42.9%) of included patients required further surgical treatment following recurrence or a failure to achieve remission after initial treatment. This demonstrates the cyclical nature of chronic infection and reinforces the need for rigorous and frequent follow-up in order to detect and successfully manage recurrences that may occur. 

Previous research has demonstrated a significant economic burden associated with the treatment of chronic osteomyelitis. Shirley et al. estimate a mean treatment cost per patient of £17,031, whilst Geurts et al. suggest a higher figure of €24,481 [5,19]. Despite a relatively small sample size, our cost analysis also reveals a significant financial burden, with a mean overall treatment cost of £39,249.50. This significant financial burden is further demonstrated by additional data on a separate cohort of 20 patients treated for chronic osteomyelitis at our centre between 2018-2019, which reveals a similar mean cost per of £35,475. Interestingly, there appears to be a large discrepancy between this high cost for the hospital and the amount of reimbursement received for treatment, with a mean deficit of £19,080.10 seen in our cohort. The complex and multiple treatments required lengthy hospital stays, and the involvement of numerous specialties is likely to contribute to the high cost per patient. This, coupled with the increasing incidence of osteomyelitis, due to an increase in the prevalence of associated risk factors such as diabetes, reflects the need for increased funding where possible [20]. Research suggests both a reduced mean length of stay and potential annual savings of over £28 million were all cases of osteomyelitis to be treated at specialist centres [4]. However, there is currently only one such specialist bone infection unit in the United Kingdom. It is therefore worth considering the need for an increase in the number of designated specialist centres nationally. One potential means of achieving this is through the creation of a hub and spoke model, as proposed by Atkins and McNally, of three to six national networks each with a single specialist centre providing multidisciplinary expertise in the treatment of bone and joint infection [21]. 

Atkins et al. suggest the use of five to six intra-operative samples for accurate pathogen identification [22]. Our case series strictly adheres to this protocol, with at least five intra-operative samples being taken in each case. However, the optimum number of tissue samples is still debated in more recent research. For example, Peel et al. suggest that the greatest accuracy is seen when three tissue specimens are taken, with no additional benefit conferred with the use of further samples [23]. On the other hand, Kheir et al. support the original suggestion by Atkins et al. of five samples, and further argue that the optimal number may depend on the type of organism identified [24]. The same study also reports that only 42.2% of cultures were positive at three days, compared to 95% at eight days, highlighting the importance of consideration of growth duration, as well as sample number [24].

We acknowledge that this study has some limitations. Firstly, although Cierny-Mader and BACH classification systems allow for a degree of standardisation, only one clinician was involved in classifying the 14 patient cases. Involving more than one clinician may have allowed for more accurate classification of disease. To the best of our knowledge, no study reporting inter- or intra-observer reliability values for the Cierny-Mader classification system exists. However, previous research has shown that the BACH classification system has a high degree of inter-observer reliability, which may reduce the need for assessment by more than one clinician in order to produce an accurate classification [25]. Secondly, whilst many previous studies largely focus on managing chronic osteomyelitis with a specific treatment protocol, due to the long time period used in our study, patients in the current series received varying surgical treatments as the favoured approach changed over time, meaning we are unable to accurately conclude the efficacy of individual treatments [26-29]. Furthermore, although our mean follow-up time of 21.4 months allows for a period of potential disease recurrence [14], the follow-up range is large (7-49 months). A longer follow-up in patients towards the lower end of this range may have allowed us to more accurately access treatment success. Lastly, due to the retrospective nature of this study, we were unable to administer the EQ-5D-5L pre-operatively. Although our comparison of scores with national population norms gives a good indication of the wider health outcomes seen after treatment for chronic osteomyelitis, this comparison does not take into account any pre-treatment functional deficits in our patients.

## Conclusions

Patients with severe grades of chronic osteomyelitis present a significant challenge to the orthopaedic surgeon. In our experience, many patients required multiple interventions, either after showing recurrence or failing to achieve remission following initial treatment. Treatment of chronic osteomyelitis is currently costly which, together with an increased incidence of the condition, demonstrates the need for better organisation of treatment resources. A hub and spoke model including designated specialist treatment centres may go some way towards facilitating this, reducing treatment costs, and potentially providing better outcomes.
